# New Jersey’s Triumph

**Published:** 1900-02

**Authors:** 


### EDITORIALS.

### NEW JERSEY'S TRIUMPH.

VETERINARY annals will record for this State one of the most remarkable gatherings ever witnessed in the history of veterinary medicine.

In one day the successful and complete amalgamation of three State societies in one; the addition of more than fifty new members at one meeting of a State society!

The surrender of two State charters and seals and the union of as strong a body of veterinarians as in any State of our Union.

The gathering together at Newark of the largest body of veterinarians that had ever assembled at one time in New Jersey.

The presence of strong veterinary representations from every section of the State.

The setting sun of the incorporation of any other State organizations by legislative action.

Such are a few of the features of this triumphant day in New Jersey, and long will it be remembered by those whose pleasure it was to be there.

This inspiring array of factional lines, this termination of fruitless efforts for supremacy has ceased, and in its stead there rises up one of the strongest bodies in any of our States. Those who gained their education in the days when schools were unknown, when only the lamp of experience burned to light the way along the darkened passages of veterinary science; those more favored in later years by college advantages, and the training of those skilled in teaching, met together to join hands in placing New Jersey in the roll of States where the profession is to lead in all that is for the welfare of the calling.

Association work will feel its valued impulse, college advancement will appreciate its encouragement, State sanitary work will be more wisely directed. The individual worth of every veterinarian will be enhanced in every community where he may reside.

The citizens and tax-payers will have burdens lightened and the people better protection from the allied diseases that wipe away the dividing lines of human and comparative pathology.

We cannot refrain from extolling in the highest the grand part in this work done by our colleague, Dr. William Herbert Lowe, of Paterson, N. J., treasurer of the American Veterinary Medical Association. Early and late, at a great expense of time and money, he covered the entire State in person and by letter, appealing to his colleagues to join him in this good movement, and when its results are fully known and appreciated there will be a united vote of thanks go out to him from every section of that State, and the good wishes and full measure of appreciation by the profession throughout America.

All success to the work of this day of days in State association work.

